# Case report: The use of netarsudil to improve corneal edema after laser peripheral iridotomy and Descemet's membrane endothelial keratoplasty

**DOI:** 10.1016/j.ajoc.2021.101087

**Published:** 2021-04-14

**Authors:** Sabrina L. Chen, Stephen A. LoBue, Himani Goyal

**Affiliations:** a New York University School of Medicine, Department of Ophthalmology, USA; b SUNY Downstate Medical Center, Department of Ophthalmology, USA

**Keywords:** DMEK, Netarsudil, Rho-kinase inhibitors, Corneal edema, LPI

## Abstract

**Purpose:**

To report a case in which netarsudil ophthalmic solution 0.02% improved refractory corneal edema after laser peripheral iridotomy (LPI) and Descemet's membrane endothelial keratoplasty (DMEK).

**Observations:**

A 63-year-old female presented with decreased vision due to corneal edema secondary to iatrogenic endothelial cell loss from previous YAG and argon laser peripheral iridotomy. Initial treatment with topical sodium chloride 5% solution was unsuccessful in resolving the edema. As a result, topical netarsudil was initiated off-label. Improvement in corneal thickness and visual acuity was noted, but after a few months, the left eye decompensated with worsening edema. Cataract surgery with DMEK was performed. Surgery was prolonged and intraoperative floppy iris was encountered. Post-operatively, the patient's best-corrected visual acuity (VA) fluctuated between 20/30 to 20/70 with persistent corneal edema. The central corneal thickness (CCT) ranged from 758 to 779 three months after surgery. Topical netarsudil was started again off-label for cornea edema once nightly. Over the next two months, visual acuity and CCT improved to 20/25 and 650, respectively. Stabilization of visual acuity and cornea edema has been maintained for eight months after initiation of topical netarsudil.

**Conclusions:**

Netarsudil, a commercially available rho-kinase inhibitor, may be an effective, non-invasive adjunctive therapy for refractory corneal edema. Our case demonstrates improvement in BCVA and CCT using topical netarsudil, which has been maintained without any vision threatening side effects.

## 1 Introduction

Netarsudil ophthalmic solution 0.02%, was approved by the U.S. Food and Drug Administration for the treatment of elevated intraocular pressure in patients with primary open-angle glaucoma. Rho-associated protein kinase (ROCK) inhibitor, such as netarsudil, act by disrupting actin fibers and decreasing smooth muscle contraction and stiffness in the trabecular meshwork, thereby increasing the flow of aqueous humor.

Interest has also arisen regarding ROCK inhibitors and endothelial function. Rho-Kinase inhibitors have been shown to improve corneal endothelial cell function by decreasing apoptosis and increasing cell proliferation in animal models.^1^^,^^2^ ROCK inhibitor Y-27632 has been studied in Fuch's endothelial dystrophy and shown to preserve corneal clarity and visual acuity.^3^ Ripasudil (K-115) has also been shown to improve corneal edema in patients after failed central descemetorhexis.^4^ To date, there is limited data on netarsudil (AR-13503), which we hypothesized would have a similar effect on endothelial cell function.^5^

## 2 Case report

**Fig. 1:**
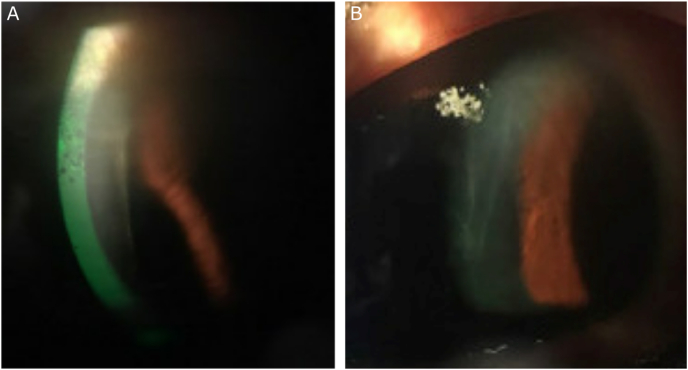
A) Superior microcystic and stromal edema extending to the visual axis in the left eye. B) Microcystic edema progressed to stromal edema with Descemet's folds in the left eye as endothelial cell loss continued.

**Fig. 2:**
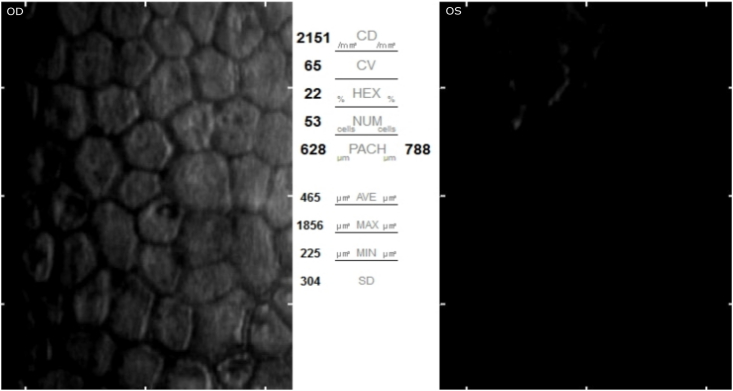
Specular microscopy showed regular endothelial cells OD with a count of 2151. No cells could be visualized OS due to the corneal edema.

A 63-year-old female patient presented for decreased vision in her left eye. Her past ocular history was significant for narrow anatomical angles, status post argon LPI in both eyes at age 54, and subsequent touch up YAG LPI in the left eye at age 58. Initial BCVA was 20/20 OD and 20/60 OS. On exam, there was edema of the superior cornea extending to the visual axis in both eyes (OU) with left eye (OS) (Fig. 1A) greater than the right (OD). There were no signs of Fuchs endothelial dystrophy. The anterior chamber was narrow and stable compared to previous exams, with no signs of inflammation. The iris was convex and the angle was open to trabecular meshwork (TM) in all quadrants on gonioscopy. The intraocular pressure (IOP) was 18. On optical biometry, anterior chamber depth was 2.55 mm OU, and central corneal thickness (CCT) was 607 OD, 747 OS. Specular microscopy showed regular endothelial cells OD with a count of 2151. No cells could be visualized OS due to the corneal edema [Fig. 2].

Our initial differential diagnosis included corneal edema from possible intermittent angle closure, endotheliitis, and most likely, iatrogenic endothelial cell loss from previous LPIs. Treatment for these modalities was initiated. NaCl 5% solution was started at four times per day dosing. The patient returned two weeks later showing no improvement. Prednisolone acetate 1% was added for a possible inflammatory component. With still no improvement, valacyclovir was started for possible herpetic endotheliitis.

Visual acuity and CCT in the right eye remained stable at 20/20 and 607, however, the left eye worsened to 20/80 and 762. Intraocular pressure (IOP) ranged from 11 to 18 in both eyes. A trial of netarsudil 0.02% once nightly was initiated, off-label, in the left eye for worsening edema. Over the next four weeks, BCVA OS improved to 20/30 with a decrease in CCT to 677 OS. IOP ranged from 11 to 16. Over the next two months, the right eye remained stable but the left eye decompensated with worsening corneal edema. Netarsudil was increased to two times a day, but there was no further improvement. CCT increased to 758 with worsening BCVA of 20/80 OS, while the IOP remained stable. Microcystic edema progressed to stromal edema with Descemet's folds in the left eye (Fig. 1B).

The patient underwent combined cataract extraction (CE) with posterior chamber intraocular lens placement and Descemet's membrane endothelial keratoplasty (DMEK) OS. Surgery was prolonged and intraoperative floppy iris was encountered. Pathology revealed a host Descemet's membrane that was devoid of endothelial cells, likely from chronic narrow angle, and iatrogenic cell loss from the LPI and cataract surgery.

**Fig. 3:**
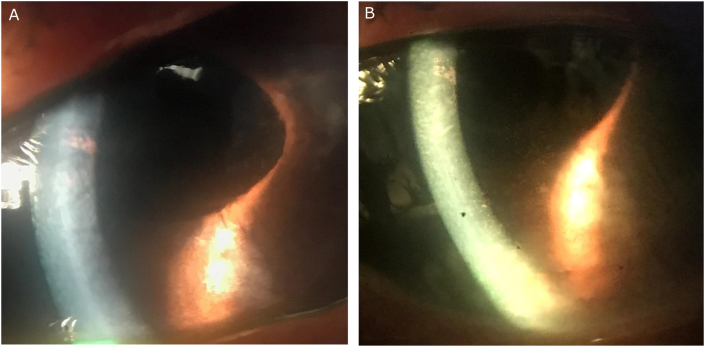
A) Persistent stromal edema several weeks after successful re-bubbling of the inferior DMEK graft. B) Interval improvement of cornea edema two months after topical netarsudil.

Re-bubbling was required at three weeks postoperatively due to a partial detachment of the inferior DMEK graft. Despite a successfully attached Descemet's membrane graft, the patient's visual acuity fluctuated between 20/30 and 20/70, CCT ranged from 758 to 779, and IOP ranged between 13 and 18, for three months after surgery (Fig. 3A). Netarsudil 0.02% was started once nightly again. Over the next two months, BCVA improved from 20/70 to 20/25, CCT improved from 779 to 650, and IOP was maintained between 11 and 17 (Fig. 3B). Continuing daily netarsudil dosing, the patient's BCVA and CCT has remained stable for eight months.

## 3 Discussion

Many ophthalmologists in the US are using netarsudil anecdotally to treat corneal edema; however, there are limited publications on its successful use without vision changing side effects like reticular bullous epithelial edema.^5^, ^6^, ^7^ We provide a detailed timeline for one patient in whom it resulted in decreased corneal thickness and improved visual acuity in two very different clinical scenarios: endothelial cell loss after LPI and delayed recovery after DMEK surgery.

The mechanism of Rock inhibitors is hypothesized to improve existing corneal endothelial cell function while minimizing endothelial loss.^1^^,^^2^ Thus, it has the potential to increase the viability and function of remaining endothelial cells.

In our report, our patient had significant endothelial loss at the time of presentation, likely from YAG and argon LPI. It has been well documented that LPI can cause endothelial damage with increasing significance at one year or greater from the initial procedure.^8^ Although we believed significant endothelial cell loss occurred at this point, the inability to visualize endothelial cells on specular microscopy was likely due to poor light penetration through the corneal edema itself.

Initial use of Netarsudil likely enhanced the function of the remaining endothelial cells, resulting in decreased edema and improved BCVA. Conversely, once the patient lost a critical number of endothelial cells, the beneficial effects of netarsudil were unlikely enough to prevent decompensation, leading to worsening edema. At the time of cataract extraction and DMEK, the host demonstrated no endothelial cells, supporting the reason why topical netarsudil ceased being effective. Yet, once DMEK was successfully completed, the refractory corneal edema was improved once netarsudil was restarted. Again, alluding to hypothesis of netarsudil increasing existing endothelial function. Since we did not find a significant IOP lowering effect from netarsudil in this case - 11 to 18 prior to any netarsudil, and 11 to 17 while on the medication – lower IOP is unlikely to have contributed to the decreased corneal edema.

Similar effects of netarsudil have been seen in other case series. Wisely et al. presented the clinical course of a patient who was started on netarsudil to treat corneal stromal edema. Treatment led to improved BVCA, and CCT, as well as eventual diminishing of corneal stromal edema, despite development of 2+ bullous, epithelial edema.^6^ Similarly, LoBue et al. presented a case series of 4 patients with stromal edema, treated with Netarsudil who developed reticular bullous epithelial edema. Among these four patients, one patient had improvements in both CCT and BVCA, despite reticular changes in the inferior, paracentral cornea.^7^

We share this case to demonstrate the efficacy of netarsudil 0.02% ophthalmic solution, a commercially available rho-kinase inhibitor in the USA, in improving corneal edema in a patient with iatrogenic endothelial cell loss from LPI and prolonged DMEK surgery. This is currently an off-label use of netarsudil and we hope cases like this will encourage further reports and clinical trials to help elucidate the clinical niche for which netarsudil is useful in our cornea patients.

## Patient consent

Patient consent to publish was obtained in writing.

## Acknowledgments and disclosures

### Funding

An unrestricted grant from 10.13039/100001818Research to Prevent Blindness (RPB, NY, NY) to the Department of Ophthalmology, NYU Langone Health (NY, NY).

## Authorship

All authors attest that they meet the current ICMJE criteria for Authorship.

## Declaration of competing interest

No conflicts of interest to report.
